# Erratum

**DOI:** 10.4269/ajtmh.15-0436err

**Published:** 2020-07-01

The authors of an article previously published by the AJTMH (Monoclonal Antibodies for the Diagnosis of *Borrelia crocidurae* by Fotso Fotso and others (https://www.ajtmh.org/content/journals/10.4269/ajtmh.15-0436) draw attention to errors in Figure 1 of that article. These resulted from errors by a researcher that were missed by other authors. Specifically, a single image was inappropriately inserted to represent three different experiments. A corrected Figure 1 is shown below.

**Figure 1.**
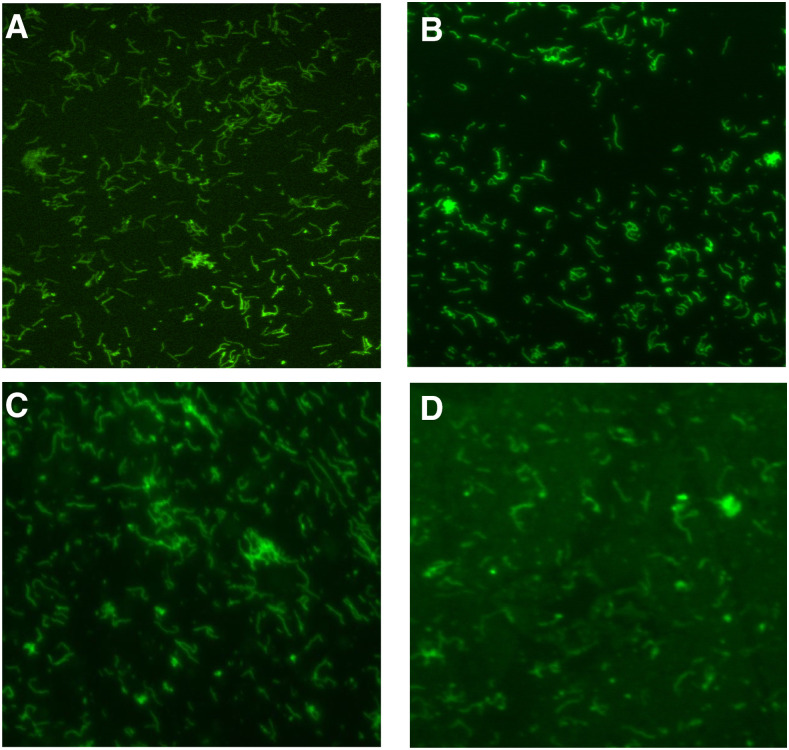
Indirect immunofluorescence assay detection of *Borrelia crocidurae* Achema strain (**A** and **B**; magnification: ×400) and clinical isolate *B. crocidurae* 03–02 (**C** and **D**; magnification: ×600) by purified mouse monoclonal antibodies (MAbs): P3A10 (**A** and **C** at 1:500 and 1:1,000 dilution, respectively) and P6D9 (**B** and **D** at 1:500 and 1:1,000 dilution, respectively). Monoclonal antibodies were tagged using a goat anti-mouse IgG conjugated to fluorescein isothiocyanate at 1:400 dilution. These two mentioned MAbs are available for research purposes upon request from the corresponding author.

